# Proposal for a Controlled Humidity Environment Test Bench for the Accurate Characterization of Icephobic Properties

**DOI:** 10.3390/mi16070756

**Published:** 2025-06-27

**Authors:** Louise Burdin, Anne-Catherine Brulez, Radoslaw Mazurczyk, Jean-Louis Leclercq, Stéphane Benayoun

**Affiliations:** 1 Ecole Centrale de Lyon, CNRS, ENTPE, LTDS, UMR5513, 69130 Ecully, France; anne-catherine.brulez@itech.fr (A.-C.B.); stephane.benayoun@ec-lyon.fr (S.B.); 2 ITECH, 69130 Ecully, France; 3 Univ Lyon, CNRS, INSA LYon, Ecole Centrale de Lyon, Université Claude Bernard Lyon 1, CPE Lyon, INL, UMR5270, 69130 Ecully, France; radoslaw.mazurczyk@ec-lyon.fr (R.M.); jean-louis.leclercq@ec-lyon.fr (J.-L.L.)

**Keywords:** anti-icing, ice adhesion strength, icing delay time, mechanical shear test, test bench, humidity

## Abstract

The accumulation of ice on equipment exposed to low temperatures raises major efficiency and safety concerns. To overcome this challenge, various strategies have been developed to create icephobic surfaces. Their characterization typically relies on the measurements of icing delay time (IDT) or ice adhesion strength. However, the absence of standardized testing equipment leads to significant variability, as each research group employs different setups and conditions. This lack of standardization complicates the comparison of results and the evaluation of surface performance. Herein, we describe the development of a new reproducible test bench that allows for simultaneous measurement of ice adhesion strength and IDT under controlled humidity conditions. Results reveal that increasing humidity leads to higher adhesion and lower IDT values. This study highlights the critical influence of humidity and suggests that tests should be performed at low humidity levels in order to accurately assess the intrinsic icephobic properties of surfaces.

## 1. Introduction

Ice accumulation on equipment exposed to low temperatures can lead to serious issues such as power line failures [1], car accidents, and flight delays or crashes [2]. Various techniques have been developed and implemented to mitigate ice accretion problems. Active methods such as piezoelectric systems [3] that generate vibrations to detach accumulated ice help to prevent ice formation, as do electrothermal solutions such as heating pads [4]; however, these solutions require repeated application, and are both costly and energy-consuming. Therefore, recent advancements in surface technologies have led to the development of icephobic materials. Often inspired by superhydrophobic surfaces, these materials aim to delay freezing and reduce ice adhesion, offering a promising alternative to conventional deicing solutions [5,6,7]. Such surfaces can be achieved through femtosecond laser texturing [8], chemical etching [9], or indirectly, for instance by texturing injection molds to replicate the desired patterns onto plastic parts [10,11].

**Figure 1:**
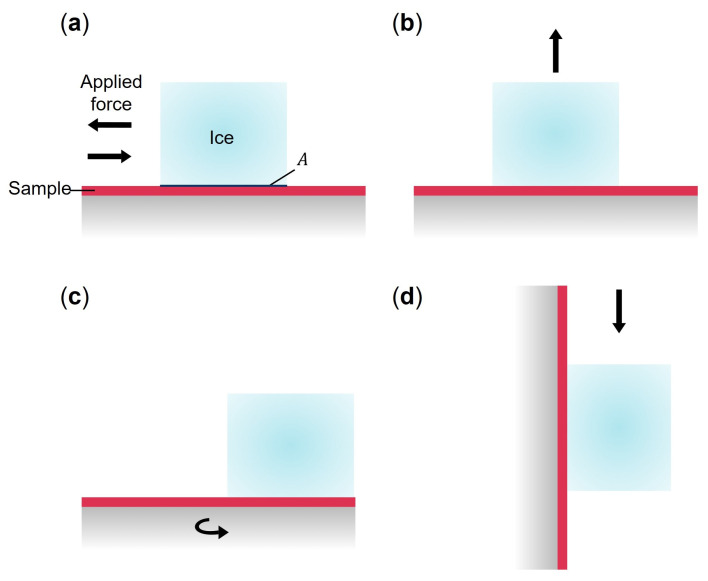
Schematic representation of the four most commonly used test methods for measuring ice adhesion strength: (**a**) horizontal shear (pull or push test) (where *A* is the contact area between the ice block and the substrate) [12,13,14,15,16]; (**b**) tensile [13,17]; (**c**) centrifuge [18,19]; and (**d**) vertical shear tests [20].

Over the past year, research on the design of surfaces capable of reducing ice adhesion has steadily increased. The ice adhesion strength τ is defined as the ratio between the maximum force *F* required to detach an ice block and the contact area *A* between the ice block and the substrate (Figure 1a):

(1) $$\tau =\frac{F}{A}.$$

**Figure 2:**
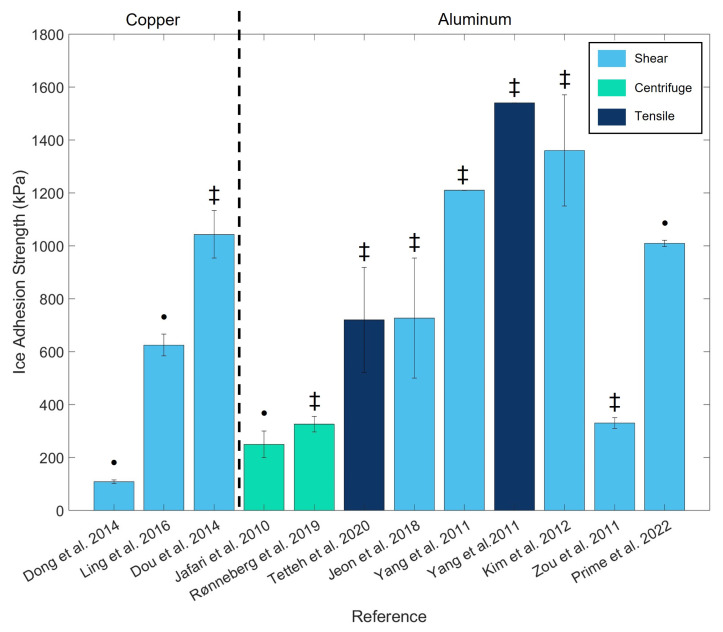
Ice adhesion measurements obtained using three different methods. Data obtained from [12,13,14,15,16,17,18,19,25,26,27]. A • indicates polished or mirror-polished surfaces, while a ‡ indicates bare or as-received surfaces.

Several methods exist for measuring F, with the four most common presented in Figure 1. However, the use of different methods for characterizing ice adhesion makes comparisons challenging. As shown in Figure 2, ice adhesion strength has a wide range of values for the same material. Moreover, the standard deviations of reported ice adhesion strengths (Figure 2) are quite high, and this disparity of results is frequently reported in the literature.

In addition to the use of different characterization methods, these disparities can also be attributed to the applied experimental conditions. Table 1 presents the data from Figure 2, focusing only on ice adhesion values obtained for aluminum using the horizontal shear test. Table 1 illustrates that each research group employs its own experimental conditions due to the lack of standardization, which can influence the obtained ice adhesion strength. In previous studies, researchers have identified specific factors that can impact ice adhesion tests. For example, Rønneberg et al. [21] highlighted the influences of ice type, test temperature, ice sample size, and distance between the force sensor and the ice block. Similarly, Bleszynski et al. [22] emphasized the role of the type of mold and the geometry used to make the ice block. Another important and frequently overlooked parameter that influences ice adhesion is the humidity. Studies that have controlled for humidity during ice adhesion tests have shown that surfaces exposed to high humidity, particularly those with significant roughness, exhibit condensation within their asperities, which can lead to increased ice adhesion [23,24]. This highlights the importance of being able to control the humidity parameter.

**Figure 3:**
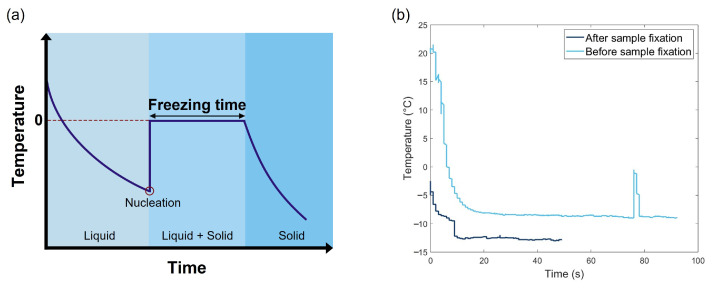
(**a**) Typical profile of water freezing and (**b**) evolution of the temperature of a distilled water droplet deposited before and after fixing a copper substrate on a cooling plate.

The icephobic properties of a surface can additionally be characterized by measuring the freezing time; however, this method also lacks standardization. For example, Guo et al. [28] measured freezing time using a charge-coupled device camera, whereas Kameya et al. [29] used an infrared (IR) camera to track the temperature evolution of a droplet over time. Additionally, in most studies the definition and measurement procedure are not clearly outlined. Figure 3a presents the typical freezing profile of water. The freezing process of a water droplet involves several stages. Initially, the temperature of the droplet decreases until it reaches values below the melting point. When the temperature becomes sufficiently low to surpass the phase transition threshold of the droplet and overcome the nucleation energy barrier, nucleation is triggered. This step also corresponds to the icing delay time (IDT). The droplet then begins to freeze, leading to a temperature increase due to latent heat release and the volume expansion associated with the phase change. This is followed by the solidification stage, which continues until the droplet is fully frozen. During this phase, the temperature remains constant (corresponding to the freezing time), which indicates the resistance of the surface to freezing. The longer this phase lasts, the higher the freezing time. After the droplet is completely frozen, the temperature decreases again until it reaches the temperature of the substrate. This decline occurs more rapidly than in the initial cooling phase owing to the higher thermal diffusivity of ice.

Thus, the development of a reproducible and controlled test bench is essential for fostering progress in this area. This study proposes the implementation of a test bench capable of characterizing the ice adhesion strength and freezing time of surfaces under humidity conditions controlled to mimic real-world environments. In the literature, ice adhesion measurements are often based on static and impact ice [17,30,31,32,33]. Static ice is formed by freezing water in a mold, while impact ice simulates rain striking a surface, introducing greater complexity and variability in ice area control. Although static ice tests do not replicate real-life ice accumulation, they provide a controlled environment to assess ice adhesion strength, aiding material development in laboratory settings. Inspired by the above features, this study focuses on static ice adhesion. Recently, several efforts have been undertaken to standardize ice adhesion tests. Among them, Wang et al. [20] proposed a vertical shear test using commercially available instruments, which helps to facilitate the reproducibility of the setup. Similarly, Irajizad et al. [34] introduced a horizontal shear test. Rønneberg et al. [21] also suggested a standardized method based on a horizontal shear test, emphasizing that vertical shear and centrifuge tests may be unsuitable for surfaces with low adhesion. Therefore, the horizontal shear test appears to be the most practical and adaptable for various surfaces. Consequently, in this study we select a horizontal shear test, specifically a pull-off test.

Initial tests were conducted on copper, aluminum, 304L stainless steel, and glass slides in order to validate the ability of the test bench to provide consistent and reproducible results. In addition, this study investigated the influence of humidity on IDT and ice adhesion.

## 2. Materials and Methods

### 2.1. Materials

To demonstrate the ability of the test bench to provide reproducible and accurate measurements, ice adhesion and IDT were measured on five samples: copper, 304L stainless steel, bare aluminum, and polished aluminum. The sample dimensions are summarized in Table 2. These samples were selected based on their varying thermal conductivity, as presented in Table 2. Furthermore, their nearly identical thickness ensures that the influence of this parameter on IDT measurements is minimized.

Before characterizing the wetting and icephobic properties of the samples, all surfaces were cleaned using ultrasound for 5 min in ethanol and for another 5 min in distilled water, followed by drying under nitrogen flow.

### 2.2. Test Bench

**Figure 4:**
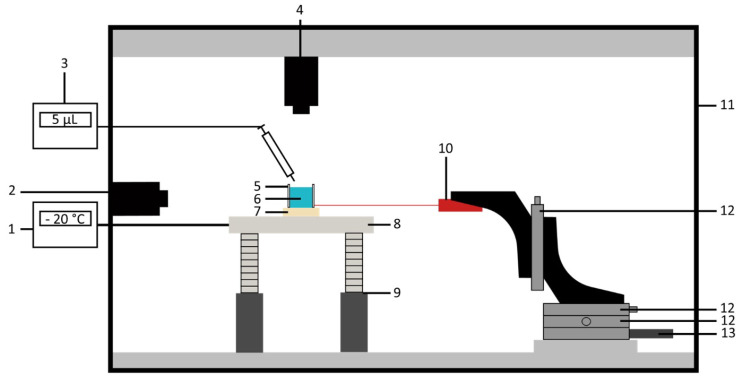
Diagram of the test bench: (1) cryostat, (2) optical camera, (3) syringe pump, (4) IR camera, (5) acrylonitrile butadiene styrene (ABS) mold, (6) ice block, (7) sample, (8) cooling plate, (9) adjustable foot for leveling, (10) force sensor, (11) glove box, (12) micrometer, and (13) motor.

**Figure 5:**
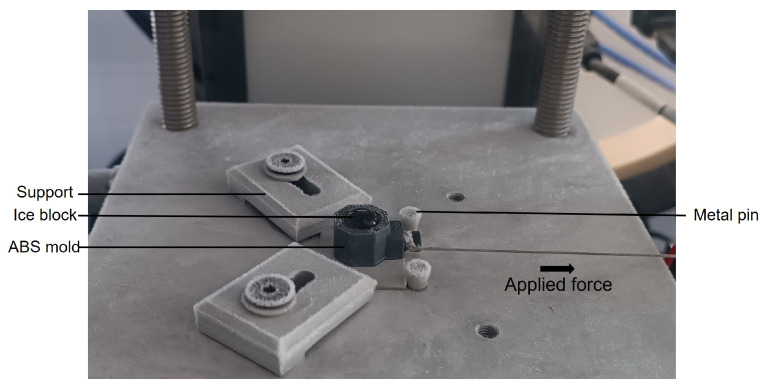
Photograph of the machined aluminum cooling plate during a horizontal shear test at 45.4 ± 0.6 %RH.

A home-made test bench for characterizing ice adhesion and freezing time was designed and built, as illustrated in Figure 4. The test bench included a machined aluminum cooling plate (8) (CTMP, Vedène, France) with temperature regulated by a cryostat (1) (FP50-ME, Julabo, Seelbach, Germany). In most studies focusing on assessing ice adhesion through the horizontal shear test, Peltier units are used to cool down the samples owing to their ease of implementation [26,32,38]. However, we chose an aluminum plate with internal cooling fluid circulation instead, as this permits integration of fastening elements for improved sample stability during adhesion measurements. As shown in Figure 5, supports can be screwed onto the plate to hold the sample in place. Additionally, metal pins enhance stability by preventing substrate movement, ensuring accurate adhesion force measurements.

During ice adhesion measurements, a force sensor (10) (LSB201, Futek, Irvine, CA, USA) records the maximum force required to detach an ice block from the surface. This sensor is controlled by a motor (13) (LTA-HL, Newport, Irvine, CA, USA), which is operated through a custom-developed LabVIEW interface. This interface also provides access to the data collected by the optical camera (2) (GOX-5102C-USB, JAI, Copenhagen, Denmark), enabling real-time monitoring of the ice detachment process.

For freezing time measurements, an IR camera (4) (Xi 400, Optris, Berlin, Germany) monitors the temperature evolution of a droplet over time. In this case, the optical camera can also be used to track the freezing front of the droplet, allowing for verification of whether or not the droplet is fully frozen. The entire test bench is placed in a glove box (11) where the humidity is controlled, primarily through pumping and introduction of dry air, enabling control of the humidity level between 18% RH and the room environment.

### 2.3. Sample Characterization

#### 2.3.1. Topography

The morphology of the samples was quantitatively assessed using an interferometer (GT-K1, Bruker, Billerica, MA, USA) in vertical scanning interferometry mode with white light illumination at 50× magnification and a 1× lens. Interferometric measurements were analyzed using MountainsMap software (version 7.4.8076, Digital Surf, Besançon, France). The arithmetic mean roughness (S_a_) was calculated using the ISO-25178 standard [39].

#### 2.3.2. Wettability

The wettability properties of the surfaces were characterized using a goniometer (DSA30, Kruss, Hamburg, Germany) following the tangent-2 method [40] in Drop Shape Analysis software (version 1.92.1.1, Kruss, Hamburg, Germany). Static contact angles (CAs) were determined using 3 µL of distilled water droplets. To obtain an estimate of the standard deviation of the measurements, five droplets were deposited in different areas of the surface.

#### 2.3.3. Ice Adhesion

The cooling plate was cooled to approximately −10 °C. An ABS mold tube with an internal diameter of 8.66 ± 0.09 mm, an outer diameter of 14.14 ± 0.12 mm, and a height of 10.13 ± 0.06 mm was then placed on the sample. A recent paper has investigated the influence of the contact area between the ice sample and the substrate on ice adhesion strength [41] and shown that the force required to detach the ice sample increases with the contact area up to a critical value of the length of the ice sample. This critical value has also been reported by Stendardo et al. [42]. Generally, two failure mechanisms can be observed, namely, stress-dominated and toughness-dominated failure. Therefore, the predominant mechanism depends on the length of the ice sample. In stress-dominated failure, interfacial rupture occurs instantaneously, whereas toughness-dominated mechanisms involve progressive crack propagation. According to Stendardo et al. [42], for small interfaces (ice sample diameters below 10 mm) failure is stress-dominated, permitting the application of Equation (1). Therefore, in this study we selected an ice block diameter of 9 mm in order to achieve sufficiently high adhesion force values while ensuring the applicability of Equation (1).

Distilled water (0.6 mL) was added to the mold tube using a syringe. The tube–sample assembly was then fixed onto the cooling plate for the simultaneous cooling of water and substrate. After the total volume was completely frozen, the wire (which was connected to the force sensor and positioned at a height of 2.43 ± 0.09 mm from the surface) was tensioned and aligned with the force sensor. Given the position of the wire, it is possible to observe torque during the ice adhesion test. The motor speed was then set to 1 mm/s and data acquisition was initiated. Equation (1) was used to determine the ice adhesion force. After the ice sample detached, the surface was visually examined to determine whether the fracture was adhesive (ice–substrate fracture) or cohesive (ice–ice fracture). Ice adhesion measurements were performed three times per sample in order to assess the reproducibility of the results while ensuring that the samples were thoroughly dried with a nitrogen flow between tests.

#### 2.3.4. IDT

The cooling plate was cooled to approximately −10 °C, then 5 µL of distilled water was gently deposited on the sample using a syringe. The sample was carefully placed on the cooling plate and an IR camera was used to monitor the temperature evolution of the droplet over time. As mentioned previously, three tests were conducted to verify the reproducibility of the results. Between each test, the sample was dried using nitrogen flow.

## 3. Results and Discussion

### 3.1. Ice Adhesion Strength Measurements

Because icephobic properties can be influenced by surface wettability properties and roughness, the S_a_ and static CAs of the samples were measured. The results are summarized in Table 3.

**Figure 6:**
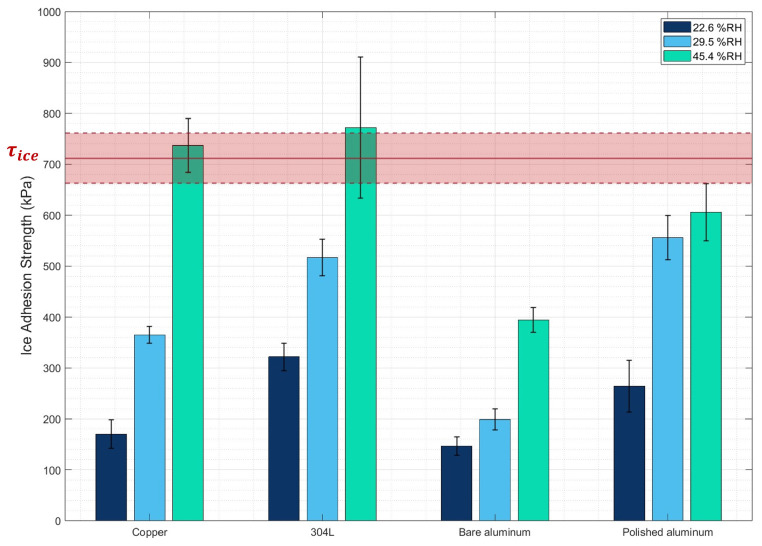
Ice adhesion strength measured on the samples at different relative humidity levels. The red box corresponds to $\tau_{ice}$ with its standard deviation.

**Figure 7:**
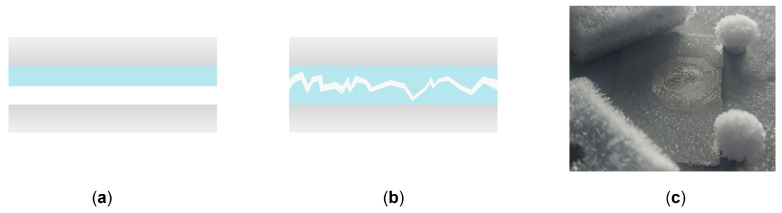
Schematic representation of (**a**) adhesive rupture and (**b**) cohesive rupture along with (**c**) a photograph of a cohesive fracture observed on 304L at 45.4 ± 0.6% RH.

To demonstrate the proof-of-concept of our test bench and its ability to produce reproducible results, ice adhesion tests were first conducted on copper, 304L stainless steel, bare aluminum, and polished aluminum at room humidity (45.4 ± 0.6% RH). Moreover, the glove box made it possible to perform adhesion measurements at different humidity levels (22.6 ± 1.1% RH and 29.5 ± 1.2% RH), allowing us to assess the influence of this parameter on ice adhesion. Figure 6 presents the obtained results, showing that as the humidity level decreases, ice adhesion also decreases. This observation is consistent with the findings of Fu et al. [23]. This can be explained by the fact that high relative humidity conditions accelerate the absolute rate of condensation [43]. The quasi-liquid layer then absorbs molecules within the microstructures, facilitating anchoring of the ice block and leading to greater adhesion. Thus, cohesive failure (ice–ice) may dominate over adhesive failure (ice–substrate) at high humidity levels (here, 45.4 ± 0.6% RH; see Figure 7). Indeed, as can be observed in Figure 7c, when the ice block detaches, some ice remains on the sample, indicating cohesive failure. To demonstrate this, the adhesion between two ice blocks ($\tau_{ice}$) was measured and found to be 712 ± 49 kPa (Figure 6), indicating that the measured adhesion corresponds more closely to ice–ice adhesion than to ice–substrate adhesion. To avoid cohesive failure and facilitate the overall comprehension of ice adhesion, it is preferable to work at low relative humidity in order to limit the presence of condensation within the microstructure, as this can provide anchoring sites for the ice block.

### 3.2. IDT Measurements

As shown in Figure 3b, the freezing behavior of a sample will differ depending on whether the droplet is deposited after the sample temperature has reached −10 °C or before the sample is fixed on the cooling plate. This phenomenon can be explained by the formation of a quasi-liquid layer on the ice surface. Surface melting takes place on the crystal surfaces of various materials at temperatures below their melting point, leading to the formation of a quasi-liquid layer. This layer acts a conducting bond, facilitating heat exchange across its surface. When the droplet and the surface are cooled simultaneously, the temperature difference between the two systems is relatively small. Consequently, thermal exchanges are prolonged; the water remains in a supercooled state for a longer period and freezes under the influence of external factors. In this case, the IDT is extended [23]. Alternatively, when the droplet is directly deposited onto a surface that has already reached −10 °C, the significant temperature difference between the two systems actually enhances the thermal conductivity efficiency of the quasi-liquid layer. As a result, the water on the surface cools rapidly, leading to loss of the specific excitation state of the quasi-liquid layer. This degradation of the layer suppresses its functional properties, resulting in a smaller IDT [23].

Moreover, as previously mentioned, freezing time measurements are not always well defined in the literature. In this study, the freezing time corresponds to the time interval during which the temperature remains constant, as observed in Figure 3a, while the IDT is defined as the time when the droplet begins to freeze. As shown in Figure 3b, the experimentally obtained curve differs slightly from the theoretical one, as the freezing time is very short; thus, it is difficult to determine the duration over which the temperature remains constant. Consequently, only the IDT is considered in this study.

**Figure 8:**
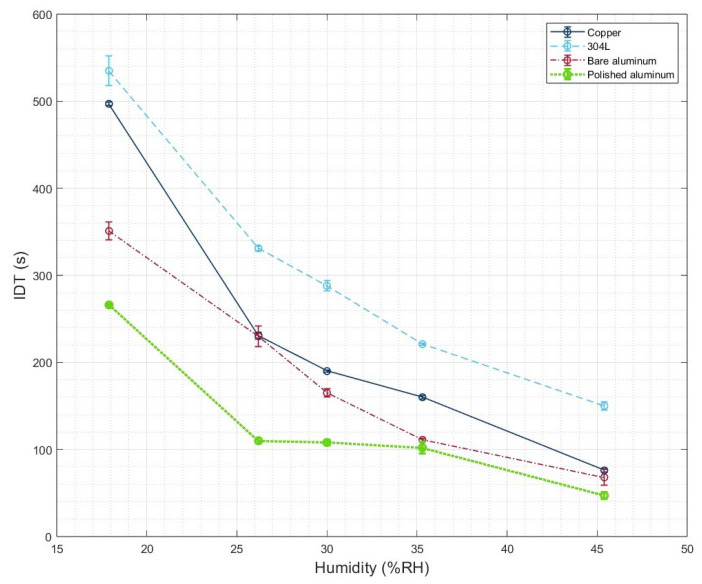
IDT measured for samples at different relative humidity levels.

Fu at al. [23] investigated the influence of humidity on ice adhesion measurements; however, they did not examine the effect of this parameter on IDT. Therefore, in this study IDT measurements were performed on different samples at room humidity (45.4 ± 0.6% RH), 17.9 ± 0.6% RH, 26.2 ± 0.7% RH, 30.0 ± 0.5% RH, and 35.3 ± 0.6% RH when using the glove box. The evolution of the IDT for each sample as a function of humidity is shown in Figure 8. It can be seen that the 304L stainless steel exhibits a higher IDT at both humidity levels. This observation can be attributed to the low thermal conductivity of 304L steel compared to copper and aluminum, which both have significantly higher thermal conductivity (Table 2).

Additionally, it can be observed that the humidity level influences the IDT; indeed, as humidity decreases, IDT increases, which is consistent with the literature. As previously stated, higher humidity levels lead to condensation. Consequently, the quasi-liquid layer absorbs the molecules present in the microstructures. This increases the contact area between the surface and the droplet, resulting in shorter IDTs. Futhermore, as shown in Figure 8, the IDT appears to be more discriminating between samples at 17.9 ± 0.6% RH than at 45.4 ± 0.6% RH. Again, to truly understand the icephobic properties of a surface, it is preferable to perform tests at low humidity levels.

Moreover, these results highlight the influence of surface roughness on IDT. According to Figure 8, the IDT values obtained for bare aluminum and polished aluminum are nearly identical at high humidity levels (>35% RH). However, at 17.9 ± 0.6% RH, the difference in IDT between these two samples becomes significantly larger. This result highlights that the influence of humidity appears to become more significant as surface roughness increases.

## 4. Conclusions

This paper describes the development of a test bench designed to characterize ice adhesion and measure IDT in an environment with controlled humidity. To verify the reproducibility of the test bench, tests were conducted on copper, 304L stainless steel, bare aluminum, and polished aluminum samples.

The test bench implements a glove box, making it possible to perform ice adhesion and IDT measurements at different humidity levels. The results of this study highlight the influence of humidity on both ice adhesion and IDT. Specifically, as the humidity increases, the ice adhesion increases but the IDT decreases. Humidity is rarely considered in the literature, despite its potential to significantly affect the results. Nonetheless, our IDT measurements indicate that the influence of humidity becomes more pronounced with increasing surface roughness and that lower humidity conditions (17.9 ± 0.6% RH) enhance the ability of the IDT to discriminate between samples. These observations support the conclusion that it is preferable to perform tests at low humidity levels in order to accurately assess the icephobic properties of a surface without interference from external factors such as condensation.

Characterizing icephobic properties is highly complex, and depends on numerous parameters; therefore, further in-depth studies are necessary in order to investigate key parameters such as substrate roughness, temperature, and ice formation conditions. Such future studies will help to refine the methodology and improve the reliability of icephobic surface characterization.

## Figures and Tables

**Table 1:** Experimental conditions used to measure ice adhesion on aluminum surfaces using the horizontal shear test (shaded boxes indicate information that was not provided in the publication; DI water refers to deionized water; Adh refers to the formula used in each publication to characterize ice adhesion).

Reference	Ice Formation	Type of Water	A (mm^2^)	Volume of Water	Velocity	Temperature (°C)	Ice Adhesion
[12]	On the test bench, until the liquid is completely frozen	DI water	79	2 mL	30 mm/min	−15	$Adh=\frac{F}{A}$
[13]	In a freezer at −10 °C, during 24 h	Water		Mold full filled	0.5 mm/min	−10	
[14]	On the test bench at −20 °C, until the liquid is completely frozen	Fresh DI water	24	150 µmL	0.5 mm/s	−10	Adh=F
[15]	On the test bench, until the liquid is completely frozen	Ultra-pure water		4 µmL droplet	1 mm/s	−10	$Adh=\frac{F}{A}$
[16]	In a freezer at −25 °C, during 3–3.5 h	DI water	36		0.33 mm/s	−10	Adh=F

**Table 2:** Sample dimensions and thermal properties.

Sample	Thickness (mm)	Width (mm)	Length (mm)	Thermal Conductivity (W·m^−1^·K^−1^)
Copper	0.92 ± 0.01	21.69 ± 0.01	32.26 ± 0.01	400 [35]
304L	0.93 ± 0.02	21.87 ± 0.03	32.25 ± 0.03	17 [36]
Bare aluminum	0.98 ± 0.01	22.11 ± 0.04	32.22 ± 0.01	237 [37]
Polished aluminum	1.59 ± 0.02	24.03 ± 0.02	32.30 ± 0.03	237 [37]

**Table 3:** S_a_ and CA values of the different samples.

Sample	S_a_ (nm)	Static CA (°)
Copper	29.2 ± 1.2	102 ± 3
304L	7.1 ± 0.3	65 ± 4
Bare aluminum	362.3 ± 6.3	96 ± 4
Polished aluminum	26.1 ± 2.8	89 ± 3

## Data Availability

All data are available within the manuscript.
